# The impact of voluntary counselling and testing services on sexual behaviour change and HIV incidence: observations from a cohort study in rural Tanzania

**DOI:** 10.1186/1471-2334-14-159

**Published:** 2014-03-22

**Authors:** Caoimhe Cawley, Alison Wringe, Emma Slaymaker, Jim Todd, Denna Michael, Yusufu Kumugola, Mark Urassa, Basia Zaba

**Affiliations:** 1 Department of Population Health, London School of Hygiene and Tropical Medicine, Keppel Street, London WC1E 7HT, UK; 2 TAZAMA Project, National Institute for Medical Research, P. O. Box 1462, Mwanza, Tanzania

**Keywords:** Voluntary counselling and testing, HIV, Sexual behaviour, Tanzania, Cohort study

## Abstract

**Background:**

It is widely assumed that voluntary counselling and testing (VCT) services contribute to HIV prevention by motivating clients to reduce sexual risk-taking. However, findings from sub-Saharan Africa have been mixed, particularly among HIV-negative persons. We explored associations between VCT use and changes in sexual risk behaviours and HIV incidence using data from a community HIV cohort study in northwest Tanzania.

**Methods:**

Data on VCT use, sexual behaviour and HIV status were available from three HIV serological surveillance rounds undertaken in 2003–4 (Sero4), 2006–7 (Sero5) and 2010 (Sero6). We used multinomial logistic regression to assess changes in sexual risk behaviours between rounds, and Poisson regression to estimate HIV incidence.

**Results:**

The analyses included 3,613 participants attending Sero4 and Sero5 (3,474 HIV-negative and 139 HIV-positive at earlier round) and 2,998 attending Sero5 and Sero6 (2,858 HIV-negative and 140 HIV-positive at earlier round). Among HIV-negative individuals VCT use was associated with reductions in the number of sexual partners in the last year (aRR Seros 4–5: 1.42, 95% CI 1.07-1.88; aRR Seros 5–6: 1.68, 95% CI 1.25-2.26) and in the likelihood of having a non-cohabiting partner in the last year (aRR Seros 4–5: 1.57, 95% CI 1.10-2.25; aRR Seros 5–6: 1.48, 95% CI 1.07-2.04) or a high-risk partner in the last year (aRR Seros 5–6 1.57, 95% CI 1.06-2.31). However, VCT was also associated with stopping using condoms with non-cohabiting partners between Seros 4–5 (aRR 4.88, 95% CI 1.39-17.16). There were no statistically significant associations between VCT use and changes in HIV incidence, nor changes in sexual behaviour among HIV-positive individuals, possibly due to small sample sizes.

**Conclusions:**

We found moderate associations between VCT use and reductions in some sexual risk behaviours among HIV-negative participants, but no impacts among HIV-positive individuals in the context of low overall VCT uptake. Furthermore, there were no significant changes in HIV incidence associated with VCT use, although declining background incidence and small sample sizes may have prevented us from detecting this. The impact of VCT services will ultimately depend upon rates of uptake, with further research required to better understand processes of behaviour change following VCT use.

## Background

Voluntary counselling and testing (VCT) for HIV has been promoted as a gateway for access to HIV treatment and care. Concurrently, the services’ potential contribution to HIV prevention has been emphasized; it is hypothesized that knowledge of HIV sero-status, accompanied by tailored and targeted risk reduction counselling, will assist both HIV-negative and HIV-positive individuals to reduce sexual risk-taking and protect themselves and their partners from HIV [1,2]. Previous studies have found mixed results with regard to the impact of VCT on reductions in sexual risk behaviour. These have generally found greater impact among individuals testing HIV-positive compared to those testing negative [3-5]. However, some studies have found no association between VCT use and changes in sexual behaviour [6,7], while others have found increases in sexual risk taking among individuals testing HIV-negative [8]. Few studies have assessed the impact of VCT on HIV incidence, however overall, these have found no significant differences between participants who received and did not receive VCT [5].

The measurement and comparison of indicators of sexual risk behaviour over time and between populations is difficult for a number of reasons, possibly explaining previous ambiguous findings regarding the impact of VCT on sexual behaviour change. Firstly, self-reported sexual behaviour data are often subject to recall and social desirability biases, the levels of which may vary depending on the behaviour in question, the amount of time that has passed since the event, and past exposure to HIV prevention messages [9]. Secondly, developing measures or indicators of sexual risk behaviour which are specific enough to accurately capture information on an individual’s sexual risk profile, yet general enough to be comparable across populations or over time, is difficult [10]. Nevertheless, while measures of sexual behaviour have not always correlated well with rates of HIV incidence or prevalence [11-13], trends in these indicators have aided our understanding of the HIV epidemic and its relationship with sexual risk behaviour [14].

In this paper, we assess the impact of VCT use on changes in reported sexual risk behaviours and HIV incidence using data from consecutive rounds of HIV serological surveillance (sero-surveys) which have been carried out as part of an on-going community cohort study in northwest Tanzania since 1994. VCT services were provided during three sero-surveys in 2003–4, 2006–7 and 2010.

## Methods

### Study setting

The study setting in Mwanza region has been described in detail by Mwaluko *et al.*[15]. Briefly, the study area lies approximately 20 km to the east of Mwanza city and consists of six villages which make up the administrative ward of Kisesa with a combined population of approximately 32,000 people, roughly half of whom are adults. The cohort consists of rounds of demographic surveillance taking place approximately once every six months since 1994, and rounds of HIV serological surveillance among adults aged 15 or older once every two to three years. HIV prevalence in the study area rose from 6.0% (95% CI 5.3% to 6.4%) in 1994–5 to 8.2% (95% CI 7.7% to 8.8%) in 2003–4 [16], before falling to 6.5% (95% CI 6.0% to 7.0%) in 2010 (R. Isingo, personal communication). This pattern may be explained by net in-migration of HIV infected individuals into the study area in the 1990’s, and a falling HIV incidence in the study area since the late 1990s [17].

Sero-surveys take place at a central point in each village, with dry-blood spot samples taken for ascertainment of HIV-status using a protocol based on informed consent without disclosure. HIV diagnosis is based on two ELIZAs (Uniform 2, Enzygnost HIV1/HIV2). A detailed questionnaire is administered by same sex interviewers, covering topics on socio-demographic characteristics, sexual behaviour and participants’ previous use of VCT services including the place and circumstances of testing, e.g. whether at a previous sero-survey or elsewhere (and if so, where).

Since the fourth sero-survey round in 2003–4 (Sero4), a separate VCT service following Tanzanian national guidelines and including pre- and post-test counselling [18] has been available to all study participants. Individuals wishing to know their HIV status are directed to a purpose-constructed hut for VCT with a trained counsellor directly after completing their questionnaire interview. At Sero4, venous blood was collected and transported to the National Institute for Medical Research (NIMR) in Mwanza, with diagnosis based on two ELIZAs as for research tests, and clients asked to return for their test results and post-test counselling one week later. During the fifth and sixth sero-survey rounds in 2006–7 (Sero5) and 2010 (Sero6) venous blood was again collected but rapid HIV screening tests were used (preliminary test using Capillus, confirmatory test using Determine. Discrepant results resolved using two ELIZAs as for research tests). Quality control was performed at the NIMR laboratory on a 5% sub-sample using two ELIZAs. During Sero5 and Sero6, HIV results and post-test counselling were usually available within 45 minutes of the rapid test being performed. Data on the uptake of VCT at sero-surveys are anonymously linked to the main sero-survey questionnaire using unique numerical participant identifiers.

Although free antiretroviral therapy (ART) was not available in 2003–4, individuals with an HIV-positive result after using VCT at Sero4 were informed that treatment would become available in the region in the near future through the national ART programme, which started at the beginning of 2005. With their prior agreement, these individuals were subsequently traced by the VCT counsellors and referred to the zonal referral hospital in Mwanza city for follow-up care. Individuals using VCT and testing HIV-positive at Sero5 were referred directly to Mwanza city hospitals. By Sero6 in 2010, a care and treatment centre was available locally at the health centre in Kisesa and so HIV-positive patients were referred here.

### Other health services in the study area

The study population is served by a government-run health centre located in Kisesa trading centre, by three small government-run dispensaries located in the rural villages, and by a number of private clinics located mainly in the trading centre. A stand-alone VCT clinic has been available at Kisesa health centre since 2005, while provider initiated testing and counselling (PITC) has been offered to all pregnant women attending the health centre for antenatal care since the end of 2008. PITC has also been available at the out-patients department since 2010, where testing may be offered to patients attending the sexually transmitted infections or tuberculosis clinics. Since mid-2009, antenatal PITC is sometimes offered to women attending the small rural dispensaries, dependant on the availability of test-kit supplies.

### Ethical statement

Ethical approval for the Kisesa cohort study has been granted by the Tanzanian Medical Research Coordinating Committee and the Ethics Committee of the London School of Hygiene and Tropical Medicine. Participation is based on informed consent without disclosure of HIV-research test results, however since Sero4 (just prior to the introduction of ART), participants are additionally offered a free VCT service as detailed above. During Sero4 verbal consent was obtained, due to low literacy rates among the study population. This was witnessed and documented for each participant on their study questionnaire, by a member of the sero-survey team. During Sero5 and Sero6 written consent was introduced (either a signature or a thumb-print, depending on the participant’s writing ability).

### Data and analysis

Nine indices of sexual behaviour were used: the number of sex partners in the last year, the acquisition or loss of spouse, regular non-cohabiting sexual partner, or high risk sex partner in the last year, whether a condom was used with spouse, regular non-cohabiting sexual partner, or high risk partner at last sex, and the coital frequency with spouse or regular non-cohabiting sexual partner in the last week. Changes in sexual behaviour were determined by comparing behaviours reported at one round with those reported at the next, in order to determine whether there had been an increase, decrease or no change in behaviour. For quantitative variables (number of sexual partners, coital frequency), differences in the constructed behaviour change variables greater than zero indicated an increase in sexual risk behaviour while values less than zero indicated a decrease and a value of zero indicated no change. For changes in binary variables (condom use at last sex), the indices represented the direction of change in behaviour, with 1 representing increased risk, 0 no change and -1 decreased risk. Multinomial logistic regression was used to assess the association between VCT and each of the nine indices of sexual behaviour change. Crude associations were adjusted for socio-demographic variables (age, sex, marital status, area of residence, level of education and reported previous use of VCT) in multivariable analyses.

In order to explore any potential differences over time, analyses were carried out separately for those a) attending both Sero4 and Sero5 and using the VCT service at Sero4, b) attending both Sero5 and Sero6 and using the VCT service at Sero5. Analyses were restricted to the non-virgin population (those who reported having ever had sex) and considered separately for individuals testing HIV-negative and HIV-positive at the time of going for VCT.

HIV incidence was estimated based on the number of initially HIV-negative respondents who seroconverted between Seros 4 and 5, and Seros 5 and 6, respectively. Dates of sero-conversion were assigned based on the midpoint between sero-survey interview dates. Poisson regression models were used to calculate crude and adjusted HIV incidence rates comparing VCT users and non-users.

## Results

### Descriptive characteristics

In total 8,961 participants attended Sero4 (participation rate of 66% [16]), of whom 3,923 (43.8%) also attended Sero5. Of these, 3,613/3,923 (92.1%) reported that they had ever had sex. 933/3,613 (25.8%) individuals expressed a desire for VCT while 324/3,613 (9.0%) actually returned to receive their results and complete VCT. In total 8,696 participants attended Sero5 (participation rate of 61% [17]), of whom 3,509 also attended Sero6 (40.3%), with 2,998/3,509 (85.4%) reporting having ever had sex. Of these, 525/2,998 (17.5%) individuals expressed a desire for VCT while 532/2,998 (17.7%) actually completed VCT. The descriptive characteristics of the non-virgin population attending the two sets of sero-survey rounds are shown in Table 1. The largest proportions of attendees were female and fell in the ≥45 age-group. Of those attending Sero4 and Sero5, 139/3,613 participants (3.8%) were HIV-positive, while 23/324 (7.1%) of those who used VCT at Sero4 were HIV-positive. Of those attending Sero5 and Sero6, 140/2,998 (4.7%) were HIV-positive and 29/532 (5.4%) of those who used VCT at Sero5 were HIV-positive.

**Table 1:** Characteristics (at earlier round) of individuals attending i)Sero-4 and Sero-5, ii)Sero-5 and Sero-6, by VCT use at earlier round

		**Attended Sero4 and Sero5**			**Attended Sero5 and Sero6**		
		**No VCT**	**Had VCT**	**Total**	**No VCT**	**Had VCT**	**Total**
		**N (%)**	**N (%)**		**N (%)**	**N (%)**	
**Total**		**3289 (91.0)**	**324 (9.0)**	**3613 (100)**	**2466 (82.3)**	**532 (17.7)**	**2998 (100)**
Sex	Male	1228 (87.1)	182 (12.9)	1420 (100)	795 (78.5)	218 (21.5)	1013 (100)
	Female	2061 (93.6)	142 (6.4)	2203 (100)	1671 (84.2)	314 (15.8)	1985 (100)
Age	15-24	815 (92.1)	70 (7.9)	885 (100)	480 (80.8)	114 (19.2)	594 (100)
	25-34	731 (86.7)	112 (13.3)	843 (100)	549 (78.1)	154 (21.9)	703 (100)
	35-44	673 (89.4)	80 (10.6)	753 (100)	471 (77.2)	139 (22.8)	610 (100)
	> = 45	1070 (94.5)	62 (5.5)	1132 (100)	966 (88.5)	125 (11.5)	1091 (100)
HIV status	Negative	3173 (91.3)	301 (8.7)	3474 (100)	2355 (82.4)	503 (17.6)	2858 (100)
	Positive	116 (83.5)	23 (16.5)	139 (100)	111 (79.3)	29 (20.7)	140 (100)
Reported previous use of VCT^$^*	No	3046 (92.2)	258 (7.8)	3304 (100)	1667 (88.9)	208 (11.1)	1875 (100)
	Yes	243 (78.6)	66 (21.4)	309 (100)	728 (69.7)	317 (30.3)	1045 (100)
Number of sexual partners in last year	None	392 (97.5)	10 (2.5)	402 (100)	490 (92.3)	41 (7.7)	531 (100)
	One	2195 (91.8)	195 (8.2)	2390 (100)	1755 (81.8)	390 (18.2)	2145 (100)
	Two or more	702 (85.5)	119 (14.5)	821 (100)	221 (68.6)	101 (31.4)	322 (100)
Marital status^$β^	Never married	435 (88.8)	55 (11.2)	490 (100)	294 (81.4)	67 (18.6)	361 (100)
	Marr monogamous	1946 (90.9)	195 (9.1)	2141 (100)	1392 (80.7)	332 (19.3)	1724 (100)
	Marr polygamous	303 (91.0)	30 (9.0)	333 (100)	268 (81.2)	62 (18.8)	330 (100)
	Widowed				276 (92.9)	21 (7.1)	297 (100)
	Separated/divorced				236 (82.5)	50 (17.5)	286 (100)
	Widowed/Separated	568 (93.4)	40 (6.6)	608 (100)			
Regular non-cohabiting partner in last year^$^	No	2776 (92.0)	242 (8.0)	3018 (100)	2130 (83.7)	415 (16.3)	2545 (100)
	Yes	513 (86.2)	82 (13.8)	595 (100)	331 (74.2)	115 (25.8)	446 (100)
High risk partner in last year^$^	No	3185 (91.3)	302 (8.7)	3487 (100)	2297 (83.0)	471 (17.0)	2768 (100)
	Yes	104 (82.5)	22 (17.5)	126 (100)	157 (73.7)	56 (26.3)	213 (100)
Ever used a condom^$#^	No	2907 (92.6)	231 (7.4)	3138 (100)	1778 (81.9)	393 (18.1)	2171 (100)
	Yes	380 (80.3)	93 (19.7)	473 (100)	133 (64.3)	74 (35.7)	207 (100)

^$^Small numbers of missing data (<3%).

*This was previous VCT use as reported at the later round (to account for any VCT use in the inter sero-survey period).

^β^Data on widowed/separated not broken down at Sero4.

^#^At Sero5 this variable related to ever use of condoms with spouse or regular non-cohabiting partners only (N = 2,403).

Across both sets of rounds, approximately 60% of attendees were married monogamously with a further 10% being in polygamous marriages. Most participants reported having one sexual partner (in the last year), and not having any regular non-cohabiting or high risk partners (also in the last year - Table 1). Condom use was rare, with just 7.4% reporting ever using a condom at Sero4, and 9.2% reporting ever using a condom with the three most recent sexual partners at Sero5.

### Impact of VCT on sexual behaviour change among HIV-negative individuals

Among HIV-negative individuals, VCT use was associated with some changes in sexual behaviour in terms of the number and type of partnerships formed, but less so in terms of condom use or coital frequency (see Tables 2 and 3). Between Sero4 and Sero5, compared to those who did not have VCT, VCT users had an increased likelihood of reducing their number of sexual partners in the last year (Table 2: adjusted risk ratio [aRR] 1.42, 95% confidence interval [CI] 1.07-1.88). A similar pattern was seen in terms of reduction in the number of sexual partners between Sero5 and Sero6 (Table 3: aRR 1.68, 95% CI 1.25-2.26).

**Table 2:** Change in sexual behaviour between Sero4 and Sero5 associated with VCT use at Sero4, HIV-negative individuals

	**N with outcome (%)**					
	**No VCT**		**Had VCT**		**Crude risk ratio (95% CI)**	**Adjusted risk ratio (95% CI)**^**¶**^
**Number of sex partners in last year**						
	**3173**	**%**	**301**	**%**		
No change	2060	64.9	169	56.1	1	
Increase	306	9.6	25	8.3	1 (0.64-1.54)	0.82 (0.52-1.30)
Decrease	807	25.4	107	35.5	1.62 (1.25-2.09)***	1.42 (1.07-1.88)**
**Acquired or lost a spouse**^**α**^						
	**3136**	**%**	**297**	**%**		
No change	2701	86.1	233	78.5	1	1
Acquired	255	8.1	43	14.5	1.95 (1.38-2.77)***	1.88 (1.29-2.74)***
Lost	180	5.7	21	7.1	1.35 (0.84-2.17)	1.56 (0.95-2.55)*
**Acquired or lost a regular non-cohabiting partner**						
	**3168**	**%**	**301**	**%**		
No change	2601	82.1	226	75.1	1	1
Acquired	251	7.9	27	9.0	1.24 (0.81-1.88)	0.88 (0.55-1.43)
Lost	316	10.0	48	15.9	1.75 (1.25-2.44)***	1.57 (1.10-2.25)**
**Acquired or lost a high risk partner**						
	**3156**	**%**	**299**	**%**		
No change	2879	91.2	267	89.3	1	1
Acquired	193	6.1	19	6.4	1.06 (0.65-1.73)	0.97 (0.58-1.62)
Lost	84	2.7	13	4.3	1.67 (0.92-3.03)	1.41 (0.75-2.65)
**Condom use at last sex with spouse**^**#**^						
	**2000**	**%**	**190**	**%**		
No change	1951	97.6	184	96.8	1	1
Stopped using	31	1.6	2	1.1	0.68 (0.16-2.88)	0.45 (0.10-1.93)
Started using	18	0.9	4	2.1	2.36 (0.79-7.04)	1.79 (0.56-5.67)
**Condom use at last sex with regular non-cohabiting partner**^**#**^						
	**179**	**%**	**31**	**%**		
No change	147	82.1	17	54.8	1	1
Stopped using	12	6.7	8	25.8	5.76 (2.07-16.08)***	4.88 (1.39-17.16)**
Started using	20	11.2	6	19.4	2.59 (0.92-7.35)	2.21 (0.60-8.14)
**Condom use at last sex with high risk partner**^**#**^						
	**12**	**%**	**5**	**%**		
No change	8	66.7	3	60.0	1	-
Stopped using	1	8.3	1	20.0	2.67 (0.12-57.62)	-
Started using	3	25.0	1	20.0	0.89 (0.06-12.25)	**-**

^¶^All outcomes adjusted for age, sex, marital status, area of residence, level of education and previous VCT use as reported at the later sero-survey round.

*p ≤ 0.1, **p ≤ 0.05, ***p ≤ 0.001.

^α^‘Acquired or lost spouse’ was not adjusted for marital status (as this was the outcome).

^#^Analyses conduced among those with relevant partner type at both rounds only.

**Table 3:** Change in sexual behaviour between Sero5 and Sero6 associated with VCT use at Sero5, HIV-negative individuals

	**N with outcome (%)**					
	**No VCT**		**Had VCT**		**Crude risk ratio (95% CI)**	**Adjusted risk ratio (95% CI)**^**¶**^
**Number of sex partners in last year**						
	**2336**	**%**	**502**	**%**		
No change	1732	74.1	338	67.3	1	1
Increase	272	11.6	74	14.7	1.39 (1.05, 1.85)**	1.13 (0.82, 1.55)
Decrease	332	14.2	90	17.9	1.39 (1.07, 1.80)**	1.68 (1.25, 2.26)***
**Acquired or lost a spouse**^**α**^						
	**2346**	**%**	**500**	**%**		
No change	2043	87.1	423	84.6	1	1
Acquired	158	6.7	44	8.8	1.35 (0.95, 1.91)*	1.19 (0.82, 1.73)
Lost	145	6.2	33	6.6	1.1 (0.74, 1.63)	1.12 (0.74, 1.70)
**Acquired or lost a regular non-cohabiting partner**						
	**2350**	**%**	**501**	**%**		
No change	2017	85.8	399	79.6	1	1
Acquired	90	3.8	23	4.6	1.29 (0.81, 2.07)	1.23 (0.73, 2.07)
Lost	243	10.3	79	15.8	1.64 (1.25, 2.16)***	1.48 (1.07, 2.04)**
**Acquired or lost a high risk or casual partner**^**Ω**^						
	**2344**	**%**	**498**	**%**		
No change	2062	88.0	406	81.5	1	1
Acquired	153	6.5	48	9.6	1.59 (1.13, 2.24)**	1.28 (0.84, 1.95)
Lost	129	5.5	44	8.8	1.73 (1.21, 2.48)**	1.57 (1.06, 2.31)**
**Condom use at last sex with spouse**^**#**^						
	**1379**	**%**	**332**	**%**		
No change	1345	97.5	326	98.2	1	1
Stopped using	18	1.3	3	0.9	0.69 (0.20, 2.35)	0.64 (0.18, 2.28)
Started using	16	1.2	3	0.9	0.77 (0.22, 2.67)	0.62 (0.17, 2.29)
**Condom use at last sex with regular non-cohabiting partner**^**#**^						
	**75**	**%**	**29**	**%**		
No change	51	68.0	20	69.0	1	1
Stopped using	12	16.0	5	17.2	1.06 (0.33, 3.40)	0.70 (0.14, 3.54)
Started using	12	16.0	4	13.8	0.85 (0.24, 2.95)	0.32 (0.06, 1.63)
**Condom use at last sex with high risk or casual partner**^**#Ω**^						
	**18**	**%**	**10**	**%**		
No change	10	55.6	6	60.0	1	1
Stopped using	1	5.6	2	20.0	3.33 (0.25, 45.11)	-
Started using	7	38.9	2	20.0	0.48 (0.07, 3.09)	-
**Number times sex with spouse in last week**^**#**^						
	**1388**	**%**	**335**	**%**		
No change	384	27.7	81	24.2	1	1
Increase	296	21.3	68	20.3	1.09 (0.76, 1.56)	1.03 (0.71, 1.51)
Decrease	708	51.0	186	55.5	1.25 (0.93, 1.66)	1.3 (0.96, 1.77)
**Number times sex with regular non-cohabiting partner in last week**^**#**^						
	**75**	**%**	**29**	**%**		
No change	27	36.0	11	37.9	1	1
Increase	19	25.3	7	24.1	0.9 (0.30, 2.76)	0.83 (0.21, 3.32)
Decrease	29	38.7	11	37.9	0.93 (0.35, 2.50)	0.92 (0.27, 3.14)

^¶^All outcomes adjusted for age, sex, marital status, area of residence, level of education and previous VCT use as reported at the later sero-survey round.

*p ≤ 0.1, **p ≤ 0.05, ***p ≤ 0.001.

^α^‘Acquired or lost spouse’ was not adjusted for marital status (as this was the outcome).

^Ω^Questions related to ‘casual’ rather than ‘high risk’ partners at Sero6, however, the two categories were considered comparable.

^#^Analyses conduced among those with relevant partner type at both rounds only.

Among HIV negative individuals, there was evidence that VCT use at Sero4 was associated with both acquiring (aRR 1.88, 95% CI 1.29-2.74) and losing (aRR 1.56, 95% CI 0.95-2.55) a spouse by Sero5 (Table 2). While similar trends were seen between Sero5 and Sero6, the results were not statistically significant (Table 3 aRR for acquiring a spouse: 1.19, 95% CI 0.82-1.73; aRR for losing a spouse: 1.12, 95% CI 0.74-1.70). Between Seros 4 and 5 and also between Seros 5 and 6, VCT use at the earlier round was also significantly associated with losing a regular non-cohabiting partner by the later round (aRR Seros 4–5: 1.57, 95% CI 1.10-2.25, aRR Seros 5–6: 1.48, 95% CI 1.07-2.04). Further investigation revealed that approximately half of those who acquired a spouse by the later round were the same people who had lost a regular non-cohabiting partner (144/298 or 48.3% of those acquiring a spouse between Sero4 and Sero5, and 106/202 or 52.5% of those acquiring a spouse between Sero5 and Sero6). As a result, at least some of the spousal acquisition between rounds is likely as a result of people marrying their regular partners. Between Seros 5 and 6, VCT use was also associated with losing a high-risk or casual partner (aRR 1.57, 95% CI 1.06-2.31).

In general there was no significant impact of VCT on condom use behaviour, although VCT at Sero4 was associated with an increased likelihood of stopping using condoms with regular non-cohabiting partners by Sero5 (aRR 4.88, 95% CI 1.39-17.16). Coital frequency information was not available at Sero4, but between Sero5 and Sero6, there was no evidence for an impact of VCT on changes in coital frequency with spouses or regular non-cohabiting partners in the last week (Table 3). In addition, there was no evidence to suggest any interaction between VCT use and gender in terms of any of the sexual behaviour change outcomes.

In total 2,010 participants attended all three sero-surveys, of whom 1,876 (93.3%) reported having ever had sex. Among these, 82/1,876 (4.4%) used VCT twice (at both Sero4 and Sero5) and 1,828/1,876 (97.4%) were HIV-negative at Sero4. In crude analyses among HIV negative individuals, those using VCT twice (at Sero4 and Sero5) were more likely to report increasing their number of sexual partners (RR 2.10, 95% CI 1.06-4.14) and acquiring a high-risk partner (RR 2.13, 95% CI 1.03-4.38) between Sero4 and Sero6, compared to those who did not use VCT twice. However there were no significant impacts of VCT on any of the sexual behaviour change outcomes in adjusted analyses.

### Impact of VCT on HIV incidence

Between Sero4 and Sero5, HIV incidence among those who did not use VCT at Sero4 was 0.86 per 100 person years (95% CI 0.70-1.06), while it was 1.04 per 100 person years (95% CI 0.56-1.94) among those who used VCT. The corresponding incidence between Sero5 and Sero6 was 0.66 per 100 person years (95% CI 0.46-0.93) among those who did not use VCT at Sero5 and 0.48 per 100 person years (95% CI 0.20-1.16) among those used VCT. In crude and adjusted analyses, VCT was not significantly associated with changes in HIV-incidence between either set of sero-survey rounds (Table 4).

**Table 4:** HIV incidence among initially HIV-negative participants, by VCT use at the earlier sero-survey round

	**N**	**Person years**	**Seroconversions**	**Incidence per 100PY**	**Crude rate ratio (95% CI)**	Adjusted rate ratio (95% CI)*
**Attended Sero4 and Sero5**						
No VCT	3160	10133.8	87	0.86 (0.70-1.06)	1	1
VCT	299	959.1	10	1.04 (0.56-1.94)	1.21 (0.63-2.34)	1.13 (0.58-2.22)
**Attended Sero5 and Sero6**						
No VCT	2343	4880.1	32	0.66 (0.46-0.93)	1	1
VCT	501	1031.0	5	0.48 (0.20-1.16)	0.74 (0.29-1.90)	0.69 (0.26-1.82)

*Adjusted for age, sex and previous VCT use as at reported at the later sero-survey round.

### Impact of VCT on sexual behaviour change among HIV-positive individuals

Fewer indicators of sexual behaviour change could be assessed among HIV-positive individuals due to small sample sizes, and VCT use was not significantly associated with changes in any of these in crude or adjusted analyses (Tables 5 and 6).

**Table 5:** Change in sexual behaviour between Sero4 and Sero5 associated with VCT use at Sero4, HIV-positive individuals

	**N with outcome (%)**					
	**No VCT**		**Had VCT**		**Crude risk ratio (95% CI)**	**Adjusted risk ratio (95% CI)**^**¶**^
**Number of sex partners in last year**						
	**115**	**%**	**23**	**%**		
No change	64	55.7	13	56.5	1	1
Increase	16	13.9	0	0.0	-	-
Decrease	35	30.4	10	43.5	1.41 (0.56, 3.54)	0.88 (0.25, 3.02)
**Acquired or lost a spouse**^**α**^						
	**114**	**%**	**23**	**%**		
No change	93	81.6	19	82.6	1	1
Acquired	8	7.0	1	4.3	0.61 (0.07, 5.18)	0.64 (0.06, 7.13)
Lost	13	11.4	3	13.0	1.13 (0.29, 4.35)	2.65 (0.46, 15.23)
**Acquired or lost a regular non-cohabiting partner**						
	**114**	**%**	**23**	**%**		
No change	79	69.3	15	65.2	1	1
Acquired	20	17.5	7	30.4	1.84 (0.66, 5.12)	3.46 (0.71, 16.83)
Lost	15	13.2	1	4.3	0.35 (0.04, 2.86)	0.26 (0.02, 2.85)
**Acquired or lost a high risk partner**						
	**115**	**%**	**22**	**%**		
No change	98	85.2	17	77.3	1	1
Acquired	10	8.7	1	4.5	0.58 (0.07, 4.80)	0.63 (0.05, 8.02)
Lost	7	6.1	4	18.2	3.29 (0.87, 12.48)	3.10 (0.50, 19.31)
**Condom use at last sex with spouse**^**#**^						
	**68**	**%**	**12**	**%**		
No change	64	94.1	10	83.3	1	1
Stopped using	2	2.9	1	8.3	3.2 (0.26, 38.64)	0.99 (0.03, 34.27)
Started using	2	2.9	1	8.3	3.2 (0.26, 38.64)	-

^¶^All outcomes adjusted for age, sex, marital status, area of residence, level of education and previous VCT use as reported at the later sero-survey round.

^**α**^‘Acquired or lost spouse’ was not adjusted for marital status (as this was the outcome).

^#^Analyses conducted among those with a spouse at both rounds only.

**Table 6:** Change in sexual behaviour between Sero5 and Sero6 associated with VCT use at the Sero5, HIV-positive individuals

	**N with outcome (%)**					
	**No VCT**		**Had VCT**		**Crude risk ratio (95% CI)**	**Adjusted risk ratio (95% CI)**^**¶**^
**Number of sex partners in last year**						
	**110**	**%**	**29**	**%**		
No change	72	65.5	21	72.4	**1**	**1**
Increase	14	12.7	3	10.3	0.73 (0.19, 2.80)	0.41 (0.08, 2.12)
Decrease	24	21.8	5	17.2	0.71 (0.24, 2.10)	0.56 (0.13, 2.38)
**Acquired or lost a spouse**^**α**^						
	**111**	**%**	**29**	**%**		
No change	88	79.3	23	79.3	1	1
Acquired	3	2.7	3	10.3	3.83 (0.72, 20.22)	4.35 (0.68, 27.93)
Lost	20	18.0	3	10.3	0.57 (0.16, 2.10)	0.68 (0.17, 2.63)
**Acquired or lost a regular non-cohabiting partner**						
	**111**	**%**	**29**	**%**		
No change	90	81.1	22	75.9	1	1
Acquired	8	7.2	1	3.4	0.51 (0.06, 4.31)	0.21 (0.01, 3.25)
Lost	13	11.7	6	20.7	1.89 (0.65, 5.53)	1.59 (0.45, 5.63)
**Acquired or lost a high risk or casual partner**^**Ω**^						
	**110**	**%**	**29**	**%**		
No change	90	81.8	25	86.2	1	1
Acquired	11	10.0	3	10.3	0.98 (0.25, 3.79)	0.53 (0.10, 2.98)
Lost	9	8.2	1	3.4	0.4 (0.05, 3.31)	0.32 (0.02, 4.35)
**Condom use at last sex with spouse**^**#**^						
	**61**	**%**	**18**	**%**		
No change	56	91.8	15	83.3	1	1
Stopped using	2	3.3	1	5.6	1.87 (0.16, 22.01)	-
Started using	3	4.9	2	11.1	2.49 (0.38, 16.27)	-
**Number of times sex with spouse in last week**^**#**^						
	**62**	**%**	**19**	**%**		
No change	18	29.0	2	10.5	1	1
Increase	14	22.6	3	15.8	1.93 (0.28, 13.16)	3.37 (0.26, 44.11)
Decrease	30	48.4	14	73.7	4.2 (0.85, 20.65)	7.55 (0.84, 67.63)

^¶^All outcomes adjusted for age, sex, marital status, area of residence, level of education and previous VCT use as reported at the later sero-survey round.

^α^‘Acquired or lost spouse’ was not adjusted for marital status (as this was the outcome).

^Ω^Questions related to ‘casual’ rather than ‘high risk’ partners at Sero6, however, the two categories were considered comparable.

^#^Analyses conducted among those with a spouse at both rounds only.

However, for some indicators, the measures of effect seemed to go in opposite directions between Seros 4 and 5 and Seros 5 and 6. For example, a larger proportion of HIV-positive individuals using VCT at Sero4 lost a spouse by Sero5 (13.0% versus 11.4%, aRR 2.65, 95% CI 0.46-15.23) and acquired a regular non-cohabiting partner by Sero5 (30.4% versus 17.5%, aRR 3.46, 95% CI 0.71-16.83) compared to those not using VCT. The opposite effect was seen between Seros 5 and 6. Compared to those not using VCT, HIV-positive individuals using VCT at Sero5 were more likely to acquire a spouse (10.3% versus 2.7%, aRR 4.35, 95% CI 0.68-27.93) and to lose a non-cohabiting partner (20.7% versus 11.7%, aRR 1.59, 95% CI 0.45-5.64) by Sero6. However, the confidence intervals around all of these estimates are very wide.

In crude analyses, VCT use at both Sero4 and Sero5 seemed to be associated with an increased likelihood of both stopping and starting using condoms at last sex with spouse by the next round (Tables 5 and 6). However, the numbers contributing to these analyses were very small (six participants between Seros 4 and 5, eight participants between Seros 5 and 6). As a result, the results were not statistically significant, nor was it possible to adjust for or investigate the effect of possible confounders.

Pooling the data for both sets of rounds (taking account of clustering for those who attended all three rounds) did reveal some evidence for an association between VCT use and a reduction in some sexual risk behaviours between rounds (results not shown). In these analyses, HIV-positive individuals using VCT at the earlier round seemed less likely to increase their number of sexual partners in the last year by the next round as compared to those not using VCT (aRR 0.22, 95% CI 0.05-1.01, p = 0.05).

## Discussion

In this study, the overall uptake of VCT at the first two sero-surveys during which it was offered was low (10% and 17% at Seros 4 and 5 respectively [19]). A recent estimate of ART coverage among those in need in Kisesa (based on individuals HIV-positive at Sero5) was also low (just 2% - [20]). As in previous studies, our findings with regard to the impact of VCT on sexual behaviour change were mixed, with service use associated with changes in some behaviours but not others. However, it was encouraging that reductions in the numbers and types of partnerships formed persisted across both sets of sero-survey rounds.

We found no evidence for an impact of VCT on changes in HIV incidence. While it is possible that a declining incidence in the study area overall and a small number of sero-conversions may have prevented us from detecting this, a number of other studies have similarly found no impact of VCT on changes in HIV incidence [6,21].

It was perhaps unsurprising that HIV-negative individuals were more likely to acquire a spouse after going for VCT, as young couples in sub-Saharan African are frequently advised to go for VCT before marriage. The interpretation or significance of the acquisition or loss of a spouse is somewhat different to the acquisition or loss of a high-risk partner. While acquisition of the latter may clearly be interpreted as a transition to higher risk behaviour, the acquisition of a spouse may be viewed as a shift to lower-risk behaviour as people theoretically move from a ‘sexually active but single’ state to a more stable, settled relationship. Conversely, the loss of a spouse (which might not be volitional, for example if it occurred as a result of widowhood) could be viewed as a transition to higher-risk behaviour as people leave settled relationships and return to a phase during which they may seek out new sexual partners.

It was somewhat more surprising that HIV-negative individuals using VCT at Sero4 were more likely to *lose* a spouse by Sero5 than those not using VCT. However, of the 201 HIV-negative individuals who lost a spouse between Sero4 and Sero5, 75 of these cases (37.3%) were as a result of widowhood. The observed association between VCT use and spousal loss between Seros 4–5 may be linked to widowhood through reverse causality, as individuals with sick partners may have been more likely to go and test than individuals with healthy partners. Further qualitative research may help to elucidate the reasons why people go (or don’t go) for VCT, the types of information that clients receive and the perceived importance of this information in helping people to change their sexual behaviour.

Condom use among study participants was low overall and VCT use was generally associated with little change in this behaviour. The finding that VCT use at Sero4 was associated with an increased likelihood of stopping using condoms at last sex with regular non-cohabiting partners by Sero5 is difficult to interpret, but may relate to the specific risk-profile of these non-cohabiting partners and/or whether they had also been for VCT and tested HIV-negative. A number of studies have found VCT use to be associated with increases in condom use [3,4,22] and so it was disappointing that we did not find similar results in this study. Condom use has previously been reported to be low in this part of Tanzania [23,24], and it is possible that negative attitudes towards their use persist in spite of messages shared during VCT. Low levels of condom use could also relate to anecdotal evidence of problems relating to their availability or supply.

There were no statistically significant changes in sexual behaviour associated with VCT use among HIV-positive individuals, except for a reduced likelihood of increasing the number of sexual partners in the last year in the pooled analysis. In the analyses done separately by round, for some indicators the measures of effect seemed to go in opposite directions between the two sets of rounds. For example, between Seros 4–5, individuals using VCT and testing HIV-positive seemed more likely to lose a spouse but to acquire a regular non-cohabiting partner by Sero5, compared to those not using VCT. Conversely between Seros 5–6, those testing HIV-positive seemed more likely to acquire a spouse but to lose a regular partner by Sero6. These results may have arisen simply as a result of the small sample sizes which yielded non-statistically significant results, but other factors could also have been at play. For example, antiretroviral therapy was more widely available in the study area between Sero5 and Sero6, and this may have assisted participants in regaining health and acquiring more permanent spousal partners. Sero-sorting could also have been taking place, with more people aware of their HIV-status by the later sero-survey rounds, allowing them to seek out other HIV-positive individuals with whom to form more permanent partnerships.

The limitations of this study relate to the observational nature of the data and to the pitfalls of working with self-reported sexual behaviour data, which are subject to recall and social desirability biases. We have previously shown that in Kisesa, VCT tends to attract individuals with higher risk sexual behaviours [19], and so as VCT uptake increases, the associations between VCT use and reductions in sexual risk behaviour among the general population may be weaker. In addition, we may have overstated the impacts of VCT if those who used the service were more likely to report declines in their risk behaviour compared to those who didn’t. However, the community in Kisesa has been exposed to various HIV information and education campaigns over a number of years, and so all study participants are likely to have had some degree of exposure to HIV prevention messages.

Our ability to detect changes in levels of risk behaviour was limited by lack of knowledge of the sexual risk profile and HIV-status of partners. Take for example an individual who reported one sexual partner at both rounds, but who had an HIV-negative partner at the earlier round and an HIV-positive one at the later round. In this scenario, there would have been an increase in the level of risk between rounds which our behaviour change index would have been incapable of detecting. Further analyses among the subset of married and co-habiting couples for whom partner HIV-status are available may warrant investigation.

The strengths of this study relate to the fact that our VCT use data were documented rather than self-reported, and we were able to adjust for reported use of HIV counselling and testing services outside of sero-surveys, which should have helped to reduce levels of measurement error and residual confounding.

## Conclusions

We found moderate but encouraging associations between VCT use and reductions in some sexual risk behaviours among HIV-negative participants, including reductions in the number of sexual partners in the last year, and a move to safer types of partnership. However, the overall uptake of VCT was low and we found no impacts of VCT on changes in HIV incidence. There were also no significant or consistent impacts of VCT on changes in sexual behaviour among HIV-positive individuals, but small sample sizes may have prevented us from discovering these. The impact of VCT services on sexual behaviour change and HIV prevention will ultimately depend upon rates of uptake, with further research required to better understand processes of behaviour change following VCT use.

## Abbreviations

VCT: Voluntary counselling and testing; ART: Antiretroviral therapy; PITC: Provider initiated testing and counselling; RR: Risk ratio; aRR: adjusted risk ratio; CI: Confidence interval.

## Competing interests

The authors declare that they have no competing interests.

## Authors’ contributions

CC developed the data analysis protocols, carried out the analyses and wrote the manuscript. AW, ES and BZ advised on data analysis protocols and amendments to these. JT, DM, MU, YK and BZ contributed to the conception, design and acquisition of the data. All authors critically reviewed the manuscript for important intellectual content. All authors read and approved the final manuscript.

## Pre-publication history

The pre-publication history for this paper can be accessed here:

http://www.biomedcentral.com/1471-2334/14/159/prepub
